# Trends in Rheumatoid Hand Surgery: Indications, Techniques, and Outcomes

**DOI:** 10.3390/jcm14020319

**Published:** 2025-01-07

**Authors:** Masanori Nakayama, Yasuhiro Kiyota, Soichiro Nakamura, Mitsuru Yagi

**Affiliations:** 1 Department of Orthopaedic Surgery, School of Medicine, International University of Health and Welfare (IUHW), Narita Hospital, 852 Hatakeda, Narita 286-8520, Japan; 2 Department of Orthopaedic Surgery, Keio University School of Medicine, Tokyo 160-8582, Japan

**Keywords:** rheumatoid arthritis, hand and finger deformity, ulnar deviation, joint-preserving arthroplasty, joint replacement

## Abstract

Rheumatoid arthritis (RA) causes persistent synovitis and arthritis, resulting in joint deformity and destruction throughout the body. As RA medications have evolved over the past 30 years, the surgical indications and techniques for RA joint deformities have changed. The aim of this review article is to summarize the recent trend of surgery for rheumatoid hand/finger deformities in previous reports and to present our recent surgical methods and outcomes for these deformities. A typical hand and finger deformity caused by RA is ulnar deviation, which is mainly caused by joint laxity and dislocation of the metacarpophalangeal joints, in addition to extensor tendon dislocation and/or wrist joint deformity. Although the incidence of hand/finger deformity and ulnar deviation caused by RA is decreasing due to advances in RA medications, patients with long-term RA or those with difficult-to-treat RA may still develop hand/finger deformity and ulnar deviation. If the hand/finger deformity is mild, it can be reduced manually, and conservative treatment with orthoses or splints may be required. If joint pain is severe despite good medical control of RA, or if the patient hopes to improve the appearance of the hand or fingers, surgical intervention is required. If there is only subluxation of the joints, which preserves their structure, reconstruction with only soft tissue surgery may be required. For example, for swan-neck deformity and boutonniére deformity, two of the most typical finger deformities due to RA, when the joint structures are almost intact and can be corrected manually, we opt for a surgical procedure that involves only soft tissue manipulation without the use of prosthetic implants. For ulnar deviation without joint destruction, we usually use a soft tissue-only surgical procedure. Our results have shown that the soft tissue-only surgery for ulnar deviation is as effective as joint replacement with implants. If the destruction of the joint has occurred and its dislocation/subluxation cannot be reduced manually, implant arthroplasty becomes necessary. If the joint destruction is severe, only intra-articular arthrodesis is required. In the era when RA can be controlled by medication, the hand surgeon should not overlook the change in the stage of the rheumatoid hand and should perform surgical intervention via the appropriate surgical method.

## 1. Introduction

Rheumatoid arthritis (RA) is a disease characterized by persistent synovitis that causes multiple and symmetrical arthritis in synovial joints throughout the body [1]. Among the various hand and finger deformities caused by RA, the most common is ulnar deviation, also called ulnar drift, which is caused by changes at the level of the metacarpophalangeal (MCP) joints. Although the cause of this deformity is still controversial, it is thought to be a combination of (1) deformity of the metacarpal head, (2) laxity or rupture of the collateral ligaments (especially on the radial side) of the MCP joints, (3) traction by the extensor tendon to the ulnar side (resulting in ulnar dislocation of the extensor tendon at the MCP joints), and (4) radial deviation of the wrist joint [2,3,4]. Ulnar deviation can be corrected manually when the joint destruction is mild, but it often progresses gradually and eventually becomes impossible to correct passively. In addition, the swan-neck and boutonniére (buttonhole) deformities are known to be the most common intrinsic finger deformities in patients with RA [5].

With the development of new drugs such as biologic disease-modifying anti-rheumatic drugs and Janus kinase inhibitors over the past 30 years; the establishment of treatment strategies for RA, which are known as “tight control” and “treat to target” [6]; and the fact that three-quarters of the patients with RA achieve remission or low disease activity [7], the overall number of RA surgeries, especially those involving large joints such as the knee and hip, has decreased [8]. However, surgeries on small joints such as the hand, finger, wrist, ankle, and foot have never decreased [8]. Patients’ needs for surgery have also changed. Surgery used to be required when there was persistent pain and swelling in the joints because medications alone could not control the disease activity of RA. Now that pain and swelling can be controlled with medication, cosmetic concerns or joint dysfunctions are the main reasons for surgery. Surgical techniques have changed to be minimally invasive and joint-preserving. Although the incidence of hand deformity, including ulnar deviation, has also been speculated to have decreased [9], patients with long-term RA and those with difficult-to-treat RA (D2TRA) who are refractory to medical therapy may still develop hand and finger deformity [10].

For small joints such as the hand, finger, and foot, the applicable surgical procedure varies depending on the degree of joint deformity and destruction. If the joint structure is relatively intact, joint-preserving surgery may be considered, which means only soft tissue augmentation without bone intervention. In the advanced stage, that is, when the joint destruction has progressed but the joint stability with soft tissues is preserved, a joint replacement with implants can be considered. As a next step when the joint destruction is severe and it becomes difficult to maintain joint function even with implant replacement, arthrodesis becomes the main surgical option [11]. Therefore, in small joints, it is important to assess the condition of the joint at the time and to perform surgical intervention at the appropriate time. In order to choose a less invasive surgery, it is important not to overlook the moment when the applicable surgical technique changes through detailed follow-up, and we believe that this is an important task that only surgeons treating rheumatoid arthritis can perform.

There are only a few reports that summarize the essence of the recent surgical method for RA hand/finger deformities. This review article aims to summarize the recent trend in the treatment of rheumatoid hand/finger deformities in several previous reports and to present our recent surgical methods and outcomes for these deformities, especially in the surgical procedure with only soft tissue intervention in comparison to that involving joint replacement with implants.

## 2. Conservative Treatment of the Rheumatoid Hand

The effectiveness of an individualized rehabilitation intervention for rheumatoid hand and finger deformities has been demonstrated in the previous study [12]. In this study, patients were randomized to receive only the usual care for the rheumatoid hand (including patient education on joint protection and splint fabrication only when required) or intervention and exercise therapy administered by a physical or occupational therapist. Results showed that at 12 months, the Michigan Hand Outcome Questionnaire (MHQ) scores for general assessment and for the activities of daily living and work were significantly higher in the exercise intervention group, while there were no significant differences in MCP joint deformity. This result indicates that rehabilitation intervention improves hand and finger function in the activities of daily living and work but does not improve the deformity.

Splints or orthoses for ulnar deviation, swan-neck deformity, and boutonniére deformity are effective in cases of mild deformity and joint destruction, improving the deformity and preventing extensor tendon dislocation during finger movement in ulnar deviation [13]. However, depending on the material, they are relatively bulky, and some patients have difficulty wearing them for a long period of time. Although we sometimes make splints or orthoses for our patients, they are used only during the waiting period for the planned surgery, or they are also used to prevent recurrence of ulnar deviation after surgery, rather than for long-term wear.

## 3. Surgical Treatment for Rheumatoid Finger Deformities (Excluding the Thumb)

As described above, the most common finger deformities caused by rheumatoid arthritis are the swan-neck deformity and the boutonniére (buttonhole) deformity. If the distal interphalangeal (DIP) and proximal interphalangeal (PIP) joint structures are almost intact and can be corrected manually, we opt for a surgical procedure that involves only soft tissue manipulation without the use of prosthetic implants. For swan-neck deformity, we prefer the Thompson–Littler method [14], in which the ulnar lateral band of the extensor tendon is cut proximally and pulled radially through the palmar side of the PIP joint and its edge sutured to the radial lateral band of the extensor tendon. This procedure is simple and effective. Postoperative treatment consists of applying an extension blocking splint for three weeks after surgery, and flexion exercises are allowed from the early postoperative period.

**Figure 1:**
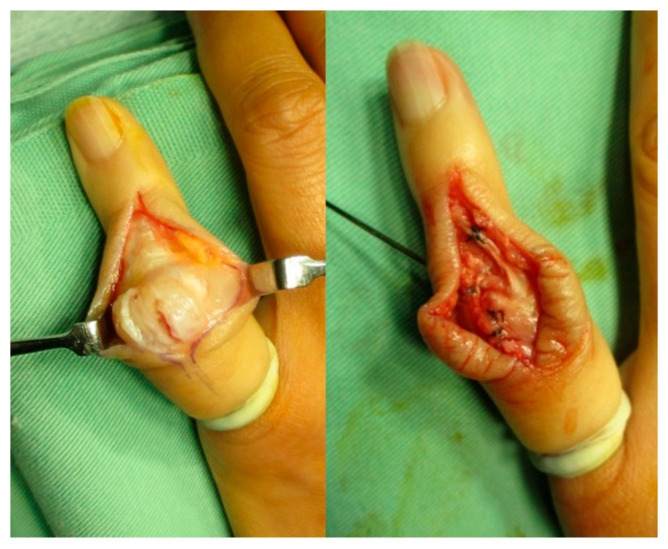
The Matev method for boutonniére deformity of the little finger. One side of the lateral band was cut and sutured to the central slip, and the other side of it was also cut and sutured to the proximal contralateral edge.

For boutonniére deformity, the Matev method is preferred [15]. This method involves cutting one side of the lateral band of the extensor tendon and suturing its edge to the central slip to increase the extension force and cutting the other side of the lateral band and suturing its edge to the contralateral proximal edge (Figure 1). It is also a simple and effective surgical procedure. Postoperative treatment consists of temporary fixation of the PIP joint with a Kirschner wire for 2 weeks, and after the wire is removed, ROM exercises are started without any restrictions. If the destruction of the PIP joint is already progressed, a joint replacement with implants is required. There are two main surgical approaches for joint replacement: the dorsal approach and the palmar approach. The dorsal approach easily facilitates the observation of the joint surfaces and the performance of procedures such as bone reaming and implant setting, while the palmar approach is a little complicated, facilitating observation of the joint surfaces to a lesser degree than the dorsal approach; however, its advantages are that the extensor tendon does not need to be cut, and postoperative joint dislocation or subluxation occurs less than in the dorsal approach [16]. Whether a constrained silicone implant or a resurfacing implant is better is still controversial. This issue of implant choice is discussed in the section “Surgical Treatment of Ulnar Deviation”.

## 4. Surgical Treatment of Rheumatoid Thumb Deformities

Nalebuff’s classification of rheumatoid thumb deformities is well known [17]. According to this classification, there are six types of deformities, including the mutilans type. Among them, type I is called buttonhole deformity, and type III is called swan-neck deformity. Types I and III are more common, while types II, IV, and V are rare. In type I, we detach the subluxated extensor pollicis longus (EPL) tendon from the MCP joint and suture it to the dorsal of the base of the proximal phalanx bone as a direct extensor to the MCP joint. Then, the extensor pollicis brevis (EPB) tendon is detached from the proximal phalanx bone, and the edge of the EPB tendon is transferred to the distal end of the detached EPL tendon as an extensor to the IP joint [18]. In moderate or severe cases, an MCP joint prosthesis is required. For type III, swan-neck deformity, suspension arthroplasty, or arthrodesis of the CM joint is required, and if the hyperextension deformity at the MCP joint has not been corrected, it should be corrected by volar capsulodesis (seasamoidesis). or arthrodesis of the MCP joint [19].

**Figure 2:**
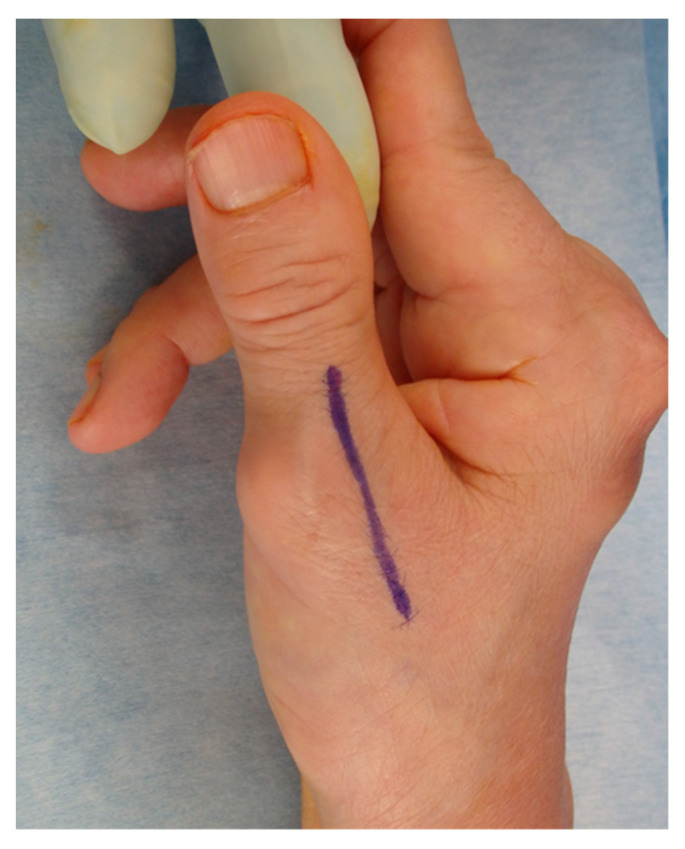
Skin incision line for the buttonhole deformity of the thumb. A longitudinal skin incision is made through 3 cm at the center of the MCP joint.

**Figure 3:**
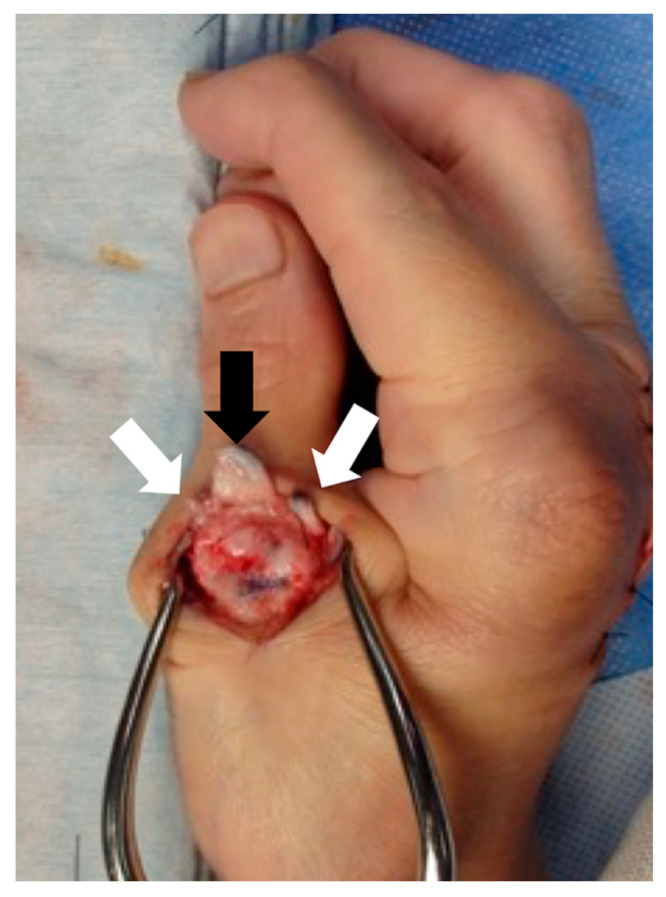
Inverted capsule and detached collateral ligaments. The capsule is cut and inverted distally (black arrow), and both the radial and ulnar collateral ligaments (white arrows) are detached from the metacarpal bone head.

**Figure 4:**
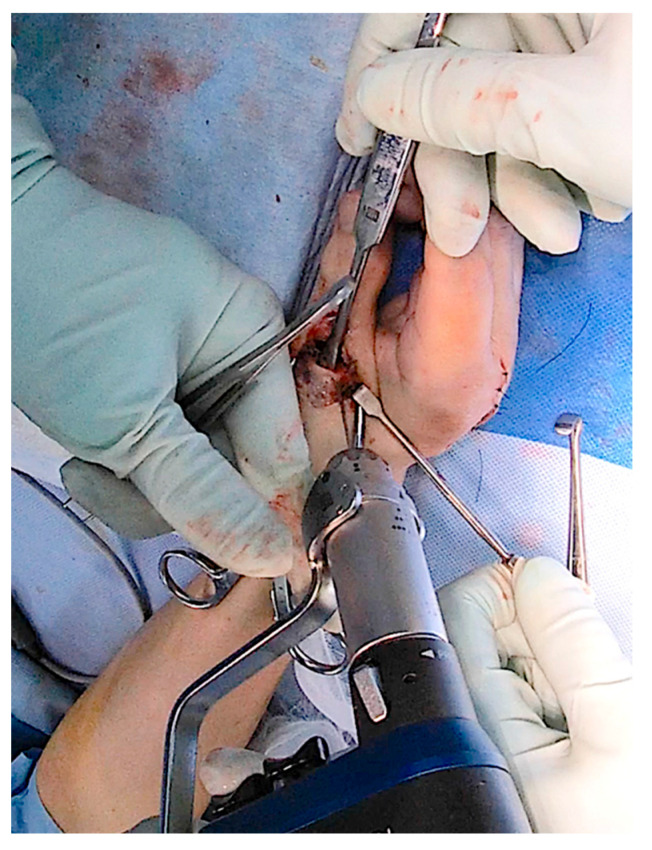
A 1.2 mm Kirschner wire is used to create two holes on both the radial and ulnar sides of the distal end of the metacarpal bone to allow the sutures for the collateral ligaments to pass through.

**Figure 5:**
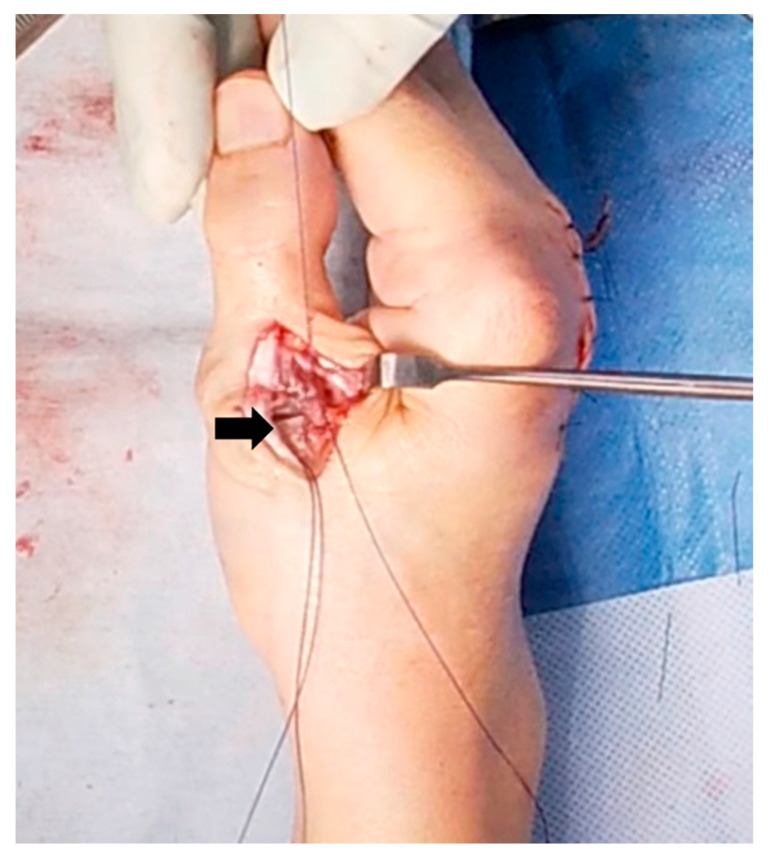
Suturing the collateral ligaments to the metacarpal bone after implant placement. After the insertion of the implants (black arrow), the radial and ulnar collateral ligaments are sutured to the metacarpal bone. In this figure, the ulnar collateral ligament is sutured using 3–0 absorbable sutures that were previously passed through the bone holes.

Our surgical technique using a dorsal approach for thumb MCP joint prosthesis is described. A skin incision is made through a 3 cm longitudinal incision at the center of the MCP joint (Figure 2), exposing the joint capsule between the EPL and EPB tendons. The capsule is cut in an arc and inverted distally while attached to the proximal phalanx bone, and both the radial and ulnar collateral ligaments are completely detached from the metacarpal bone head (Figure 3). Unlike the other fingers (described in detail below), the thumb MCP joint requires reconstruction of both the radial and ulnar collateral ligaments, so both ligaments should be carefully detached and preserved. The metacarpal bone head is resected with a bone saw. Although resection of the surface of the proximal phalanx bone is not usually necessary, if the thumb deformity is prolonged, the surface is often deformed at an angle and resection of the deformed portion is sometimes required. After testing the trial implants, two holes are created on both the radial and ulnar sides of the distal end of the metacarpal bone with a 1.2 mm Kirschner wire, and sutures (usually 3–0 absorbable sutures) are placed through each hole to attach the collateral ligament edges (Figure 4). After the insertion of the implants, the radial and ulnar collateral ligaments are sutured to the metacarpal bone (Figure 5); then, the inverted joint capsule is sutured to the bilateral collateral ligaments. In cases of severe buttonhole deformity, two holes are drilled anterior to the original position of the proximal phalanx bone, and the EPL tendon is sutured to these holes in an attempt to advance the EPL attachment site to correct the buttonhole deformity.

## 5. Surgical Treatment of Ulnar Deviation

### 5.1. MCP Joint-Preserving Arthroplasty with Soft Tissue Manipulation

As described above, in small-joint surgery, the timing of surgery and the choice of surgical technique are closely related to the condition of the joint. Therefore, it is extremely important to determine the timing of surgery. Since the drugs for RA have been developed, joint-preserving arthroplasty, which corrects ulnar deviation only with soft tissue manipulation and without bone intervention and prosthetic implants, is much more important than it was the past [20]. This section describes our surgical technique and the results of MCP joint-preserving arthroplasty for ulnar deviation.

#### 5.1.1. Indications for MCP Joint-Preserving Arthroplasty

Our subjects for MCP joint-preserving arthroplasty for ulnar deviation are patients who meet all three of the following criteria [20]:

(1) The patient has good control of RA disease activity; remission or low disease activity with a Disease Activity Score (DAS) 28 [21].
(2) Ulnar deviation can be corrected manually.
(3) The patient has a radiographic Larsen grade of 0 to 2, indicating that the joint structure is well preserved.

**Figure 6:**
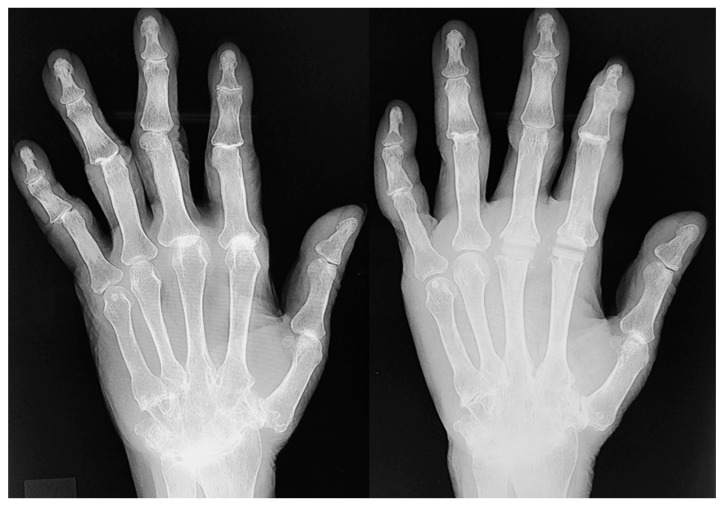
Radiographs of “hybrid finger arthroplasty” ((**left**): preoperative; (**right**): postoperative). The MCP joints of the index and middle fingers were replaced with implants, and the ring and little fingers underwent joint-preserving arthroplasty.

If even one of these criteria is not met, a joint replacement with implants is required. In cases in which these criteria are partially met, i.e., some fingers meet the criteria while others do not meet it, we perform a “hybrid finger arthroplasty” in which the fingers that meet the criteria are treated with an MCP joint-preserving arthroplasty and the others are treated with a prosthesis (Figure 6).

#### 5.1.2. Surgical Technique

**Figure 7:**
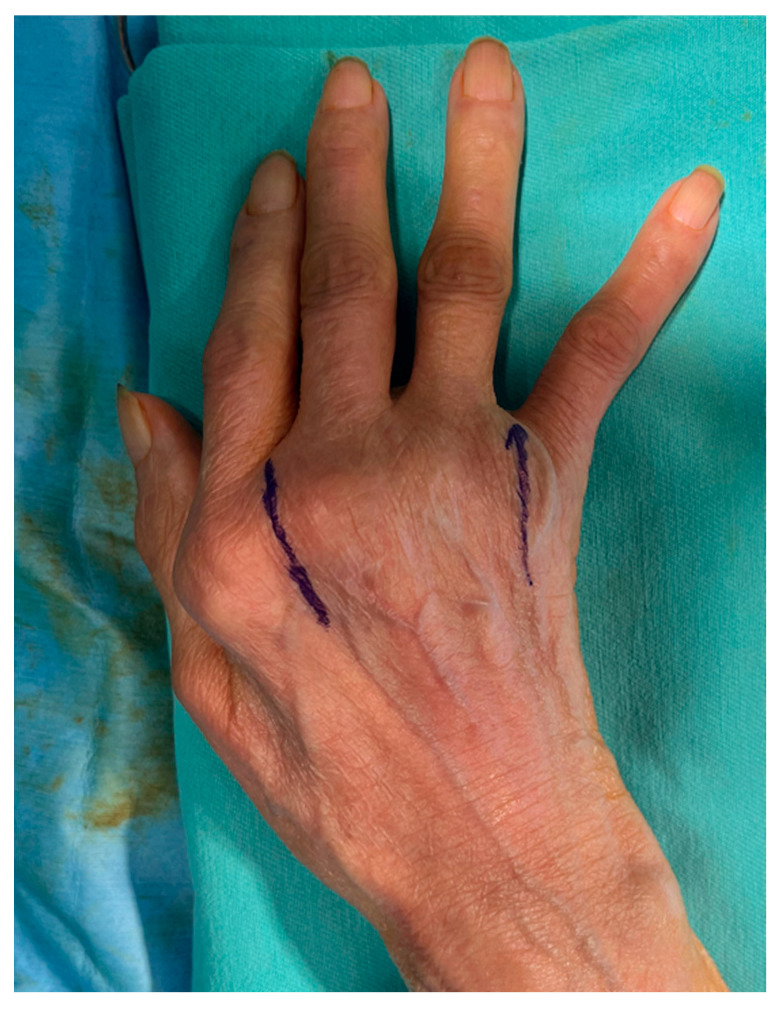
Our skin incision line for MCP joint arthroplasty. Two skin incisions are made vertically between the metacarpal heads of the index and middle fingers and between those of the ring and little fingers.

The basic procedure for MCP joint-preserving arthroplasty is the same as for the MCP joint prosthesis (described below) until the metacarpal bone head is exposed. In general, two types of skin incision lines are used in MCP arthroplasty. One is a transverse skin incision that runs horizontally across the metacarpal heads from the index finger to the little finger, and the other is two vertical skin incisions of approximately 3 cm that are made between the metacarpal heads of the index and middle fingers and between those of the ring and little fingers, respectively (Figure 7). We prefer vertical skin incision because the scars are likely to be less noticeable after surgery. The expansion hood is then revealed subcutaneously, and an incision is then made on the radial side of the dislocated extensor tendon to expose the joint capsule. The capsule is cut in an arc and inverted distally while still attached to the proximal phalanx bone, detaching the radial and ulnar collateral ligaments to fully expose the metacarpal head. If ulnar deviation is still present at this point, the contracted ulnar interosseous muscle, lumbricalis muscle, and abductor digiti minimi muscle tendons are dissected from their attachment to the proximal phalanx bone (“intrinsic release”) [11]. Suture anchors (with 3–0 non-absorbable sutures) are placed on the radial side of the metacarpal head, and only the radial collateral ligament is sutured, leaving the ulnar collateral ligament free. The inverted joint capsule is sutured to the radial collateral ligament, the extensor tendon is sutured centrally, and the expansion hood is sutured according to Wood’s procedure [22].

#### 5.1.3. Postoperative Treatment

Patients are immobilized with a splint with the MCP joint in extension and free distal to the PIP joint, and range of motion (ROM) exercises of the PIP and DIP joints are actively practiced immediately after surgery. Three weeks after surgery, the splint is removed and ROM training of the MCP joint is started.

#### 5.1.4. Results

The results of this MCP joint-preserving arthroplasty, which we have summarized in the past [20], showed that the ulnar deviation was significantly improved (using the ulnar deviation angle for evaluation, which is the angle between the axis of the proximal phalanx bone of the middle finger and that of the metacarpal bone of the middle finger [20], the average preoperative ulnar deviation angle was 28 degrees and the postoperative ulnar deviation angle was 6 degrees), and the ROM of the MCP joint was better in extension than before surgery (average preoperative extension was −20 degrees and average postoperative extension was −3 degrees), while slightly worse in flexion (average preoperative flexion was 78 degrees and average postoperative flexion was 65 degrees), with the overall arc of motion after surgery being equivalent to that before surgery (average preoperative was 58 degrees and average postoperative was 62 degrees). Preoperative pain improved in all patients, with no cases of residual pain, and there were no cases of surgical site infection or apparent recurrence of ulnar deviation during an average follow-up period of 13.5 months. There have been only a few reports on the long-term outcomes of MCP joint-preserving surgery for ulnar deviation due to RA. Wood et al. reported the long-term results for 81 months of the 16 hands in the 12 cases. In their cases, complete pain relief was achieved in 88%, the mean active metacarpophalangeal range of motion was 56 degrees, and ulnar deviation was corrected to an average of 6 degrees, which is equivalent to our results. However, swan-neck deformity progression was shown in 13 fingers and radial deviation in 2 fingers [22]. Dell et al. reported that the long-term results for 9 years of the 71 fingers in the 15 cases were good, in which the ulnar deviation was significantly improved, but 3 cases (for swan neck deformity, radial deviation and skin slough with tendon exposure) had to undergo revision surgery [3]. Given the above characteristics of postoperative ROM of the MCP joints and the possibility of such complications after long-term observation, the indication for this procedure should be considered in the context of the patient’s work and social activities.

### 5.2. MCP Joint Replacement

When joint destruction or dislocation has progressed beyond the indication for MCP joint-preserving arthroplasty, MCP joint replacement is required. Currently, there are several types of finger joint implants, which can be divided into two types: an unconstrained (surface replacement) type [23] and a constrained type [24]. There have been several reports on the long-term results of joint replacement with each type of implant. Claxton et al. reported the outcomes for 9.5 years of arthroplasty with a surface replacement prosthesis made of metal and polyethylene, and they showed good results with a high rate of pain relief and improvement of ROM and grip strength. However, at least one complication occurred in 64% of cases, but the 10-year survival rate from implant revision was 85% [25]. Goldfarb et al. reported the outcomes for 14 years of MCP replacement with a silicone implant. According to their results, MHQ, activities of daily living, satisfaction with cosmetic appearance, and overall satisfaction varied among patients, while ulnar deviation and ROM in extension improved, and ROM in flexion worsened, resulting in a postoperative arc of motion equivalent to that of the preoperative period. The implant fracture rate is 67% for Swanson implants and 52% for Sutter implants [26]. According to the reports by Parker et al. regarding pyrocarbon arthroplasty for MCP joint deformity due to RA, the results for 17 months showed that pain relief was highly achieved, and ROM and extension lag improved; however, grip strength decreased, and in radiographic outcome, axial subsidence occurred in 55% of patients, and periprosthetic erosions occurred in 45% of them [27]. To the best of our knowledge, there are no reports on the long-term results of MCP joint replacement with pyrocarbon implants in RA patients. As mentioned above, although the choice of implants is still controversial and depends on the surgeon’s preference, we have mainly used silicone implants for MCP joints, which is one of the constrained types of implants because (1) they are more likely to provide postoperative stability, even in cases with severe soft tissue destruction; (2) they are less likely to cause patient discomfort in the event of implant fracture; and (3) it is relatively easy to perform a revision surgery [11,28].

In general, for the index finger (and thumb), the stability of the MCP joint is more important than the ROM at the MCP joint in terms of being able to perform stable pinch movements, whereas for the ring and little fingers, it is important to be able to perform gripping movements, so good ROM (especially flexion) at the MCP joint is required. In previous reports, because the ROM after MCP joint arthroplasty was particularly poor in the little finger, the use of a model of the implant with pre-bending in flexion was more advantageous for the postoperative ROM in the little finger [29]. We used to use the normal type of implants for all fingers, but recently, depending on the case, we have used the pre-bent type for the little finger and sometimes for the ring finger.

#### 5.2.1. Surgical Technique

**Figure 8:**
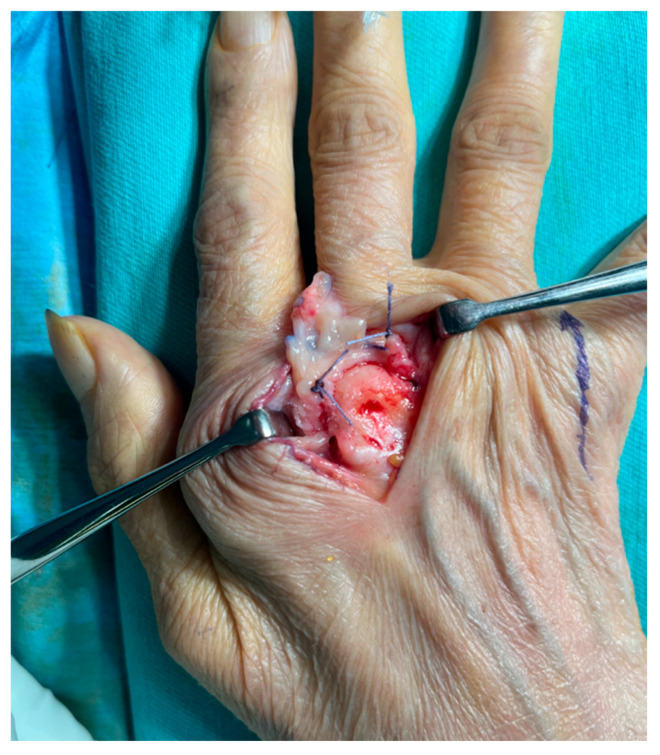
Exposed metacarpal bone head. The capsule is cut and inverted distally, and the radial and ulnar collateral ligaments (with sutures as markers) are shown.

**Figure 9:**
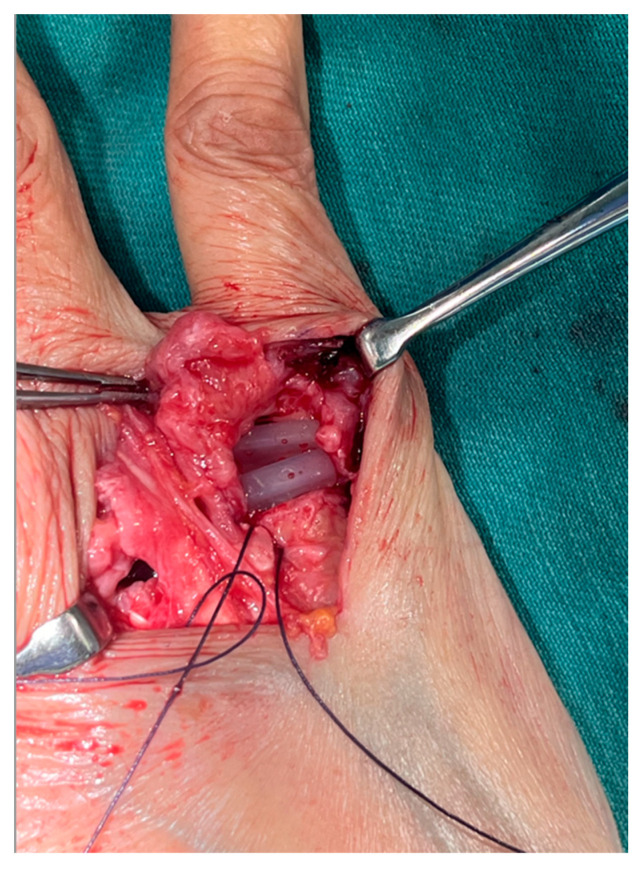
A Pre-Flex-type Sutter silicone implant is inserted into the MCP joint of the little finger. Suture threads for the later suturing of the radial collateral ligament are visible.

**Figure 10:**
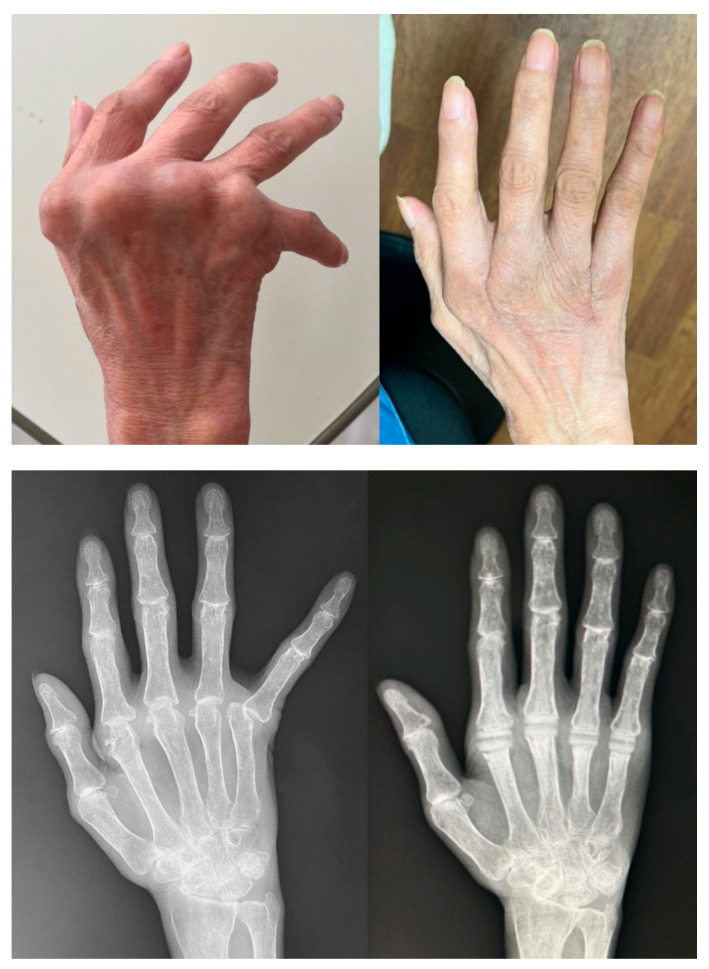
Preoperative and postoperative appearance and radiographs for MCP joint replacement with silicone implants ((**left**): preoperative; (**right**): postoperative). Ulnar deviation is improved both in appearance and in the radiographs.

The surgical technique for MCP joint replacement is the same as in the MCP joint-preserving arthroplasty described above until the metacarpal bone head is exposed. We try to preserve the joint capsule as much as possible, similar to MCP joint-preserving arthroplasty cases, although it is often significantly thinned, and sometimes its original shape may not be preserved because the joint deformity or destruction is severe compared to MCP joint-preserving arthroplasty cases (Figure 8). The metacarpal bone head is cut with a bone saw. This osteotomy should not be too small as the joint length will increase after the prosthesis insertion, resulting in increased postoperative extensor tendon tension and a disadvantageous flexion ROM. The optimal size of the osteotomy is generally 12 to 13 mm [19]. The articular surface of the proximal phalanx bone, unlike the metacarpal bone, usually does not require cutting and can be reamed without an osteotomy. If the cortex is so hard, a hole is drilled with an air drill or Kirschner wire. In cases of severe preoperative palmar dislocation of the proximal phalanx bone, where the articular surface is often obliquely deformed, a proximal phalanx bone osteotomy may be performed. Based on the reaming size of the metacarpal and proximal phalanx bones, the implant sizes are determined, and trial implants are placed. It is important to carefully insert the trial and the real implants to prevent the implants from bouncing off the operating table. If the trial implant is too tight or the ROM is too limited, a metacarpal bone osteotomy should be added. After the implant trial, two holes are drilled on the radial side of the metacarpal bone with a 1.2 mm Kirschner wire, and sutures (usually using 3–0 absorbable sutures) are passed through the holes. The same procedure is performed on the other fingers, and then the real implant is inserted (Figure 9). After implant insertion, the radial collateral ligament is sutured to the metacarpal bone using the suture previously passed through the metacarpal bone. The ulnar side is not sutured, as is the case with joint-preserving arthroplasty. The joint capsule is then sutured, and the extensor tendon is centralized. The preoperative and postoperative appearance and radiographs are shown in Figure 10.

#### 5.2.2. Postoperative Treatment

We use the same method as in the MCP joint-preserving arthroplasty; patients are administered with a splint, with the MCP joint in extension and free to move distal to the PIP joint for 3 weeks after surgery. Only in cases of high preoperative instability of the MCP joint is the orthosis for preventing recurrent ulnar deviation applied. The orthosis is removed at 4 weeks. After removal of the splint or orthosis, ROM exercises including the MCP joint are performed.

#### 5.2.3. Results

According to several reports, after MCP joint replacement with implants, a significant improvement in ulnar deviation can be expected, and the postoperative ROM is likely to decrease in flexion angle and improve in extension angle such that the arc of motion does not change significantly before and after surgery [30,31,32]. Therefore, it is necessary to explain in detail to patients who wish to undergo this surgery that the ulnar deviation will be improved, resulting in a good appearance of the hand, and the MCP joint extension will also be improved, but flexion will worsen and full grip motion may become difficult.

#### 5.2.4. Complications

Compared to non-implant arthroplasty, there are two major complications to be aware of with joint replacement. First, silicone implants are known to fracture at a high rate after surgery. As mentioned above, Goldfarb et al. reported that the fracture rate of silicone implants at 14 years was 52–67% [26], and it is reported that 26% of implants fracture early, i.e., within 3 years after surgery [28]. However, it has been reported that silicone implants do not necessarily require revision surgery, even when they rupture [33]. This may be because the remaining fractured implants still serve as joint spacers and, in many cases, do not cause major functional problems unless there is extreme dislocation or joint laxity. Another important complication is infection. The infection rate after finger joint replacement surgery is estimated to be about 0.2% [33]. Similar to infections after other joint replacements, treatment includes administration of antibiotics, local debridement and/or removal of the implant, and a two-stage revision. If removal of the implant becomes necessary, silicone implants are easier to remove than surface replacement implants. Thus, the low possibility of bone damage at the time of implant removal is a major advantage of silicone implants.

## 6. Conclusions

With the development of medical therapy for RA, the number of surgeries that are performed to reduce swelling and pain, which were important in the past, is decreasing, and more joint-preserving and minimally invasive surgeries are required. For hand/finger deformities due to RA, the appropriate surgical method, such as joint-preserving surgery, joint replacement, and arthrodesis, will change depending on the joint condition. We believe that today’s hand surgeons need to be able to choose the appropriate surgical technique for RA-related hand and finger deformity and ulnar deviation without overlooking the timing of the surgery.
